# Superficial and deep sternal wound infection after more than 9000 coronary artery bypass graft (CABG): incidence, risk factors and mortality

**DOI:** 10.1186/1471-2334-7-112

**Published:** 2007-09-23

**Authors:** Abbas Salehi Omran, Abbasali Karimi, S Hossein Ahmadi, Setareh Davoodi, Mehrab Marzban, Namvar Movahedi, Kyomars Abbasi, Mohammad Ali Boroumand, Saeed Davoodi, Naghmeh Moshtaghi

**Affiliations:** 1 Tehran Heart Center, Tehran University of Medical Science, Tehran, Iran; 2 Imam Khomeini Hospital, Infection Disease Department, Tehran University of Medical Science, Tehran, Iran

## Abstract

**Background:**

Sternal wound infection (SWI) is an uncommon but potentially life-threatening complication of cardiac surgery. Predisposing factors for SWI are multiple with varied frequencies in different studies. The purpose of this study was to assess the incidence, risk factors, and mortality of SWI after coronary artery bypass grafting (CABG) at Tehran Heart Center.

**Methods:**

This study prospectively evaluated multiple risk factors for SWI in 9201 patients who underwent CABG at Tehran Heart Center between January 2002 and February 2006. Cases of SWI were confirmed based on the criteria of the Centers for Disease Control and Prevention. Deep SWI (bone and mediastinitis) was categorized according to the Oakley classification.

**Results:**

In the study period, 9201 CABGs were performed with a total SWI rate of 0.47 percent (44 cases) and deep SWI of 0.22 percent (21 cases). Perioperative (in-hospital) mortality was 9.1% for total SWI and about 14% for deep SWI versus 1.1% for non-SWI CABG patients. Female gender, preoperative hypertension, high functional class, diabetes mellitus, obesity, prolonged intubation time (more than 48 h), and re-exploration for bleeding were significant risk factors for developing SWI (p = 0.05) in univariate analysis. In multivariate analysis, hypertension (OR = 10.7), re-exploration (OR = 13.4), and female gender (OR = 2.7) were identified as significant predictors of SWI (p < 0.05 for all). The rate of SWI was relatively similar in 3 groups of prophylactic antibiotic regimen (Cefazolin, Cefazolin + Gentamycin and Cefazolin + Amikacin: 0.5%, 0.5%, and 0.34% respectively).

**Conclusion:**

Rarely reported previously, the two risk factors of hypertension and the female gender were significant risk factors in our study. Conversely, some other risk factors such as cigarette smoking and age mentioned as significant in other reports were not significant in our study. Further studies are needed for better documentation.

## Background

Mediastinitis is an infrequent, yet potentially devastating complication following coronary artery bypass grafting (CABG). This complication is associated with amplified cost of care, prolonged hospitalization, and increased morbidity and mortality [1].

According to the Centers for Disease Control and classifications, the infection of surgical wounds of sternotomies should be considered as (A) superficial if only the skin and subcutaneous tissue are involved, (B) deep when the infection reaches the sternum but does not involve it, and (C) organ/space when sternal osteomyelitis or mediastinitis occurs. This classification enables a better comparison of related research.

For the benefit of consistency in comparing data from various reports, sternal wound infection (SWI) was sub-divided into two groups: (A) superficial SWI: wound infection confined to the subcutaneous tissue and (B) deep SWI (bone and mediastinitis): wound infection associated with sternal osteomyelitis with or without infected retrosternal space [2]. Deep SWI (bone and mediastinitis), accompanied by a high mortality rate, has an overall incidence of 0.4% to 5% independent of the type of surgery. The incidence of superficial surgical site infection in sternotomies should be similar to that in any clean surgical procedure; i.e. approximately 2%. Nonetheless, the infection rate reaches three times this value among heart disease patients on account of the fact that these patients are burdened with a high number of risk factors by comparison with the general population [2,3,13].

The present study sought to evaluate the impact of different factors on developing post-CABG SWI. The cases of deep SWI were classified in accordance with the Oakley classification of mediastinitis; there were four subtypes based on the time of first presentation, the presence or absence of risk factors, and whether previous attempts at treating the condition had failed [2].

## Methods

A total of 9201 consecutive patients who underwent isolated CABG at Tehran Heart Center, Tehran – Iran, between January 2002 and February 2006 were included in this retrospective analysis of prospectively collected data. SWI was divided into superficial SWI and deep SWI. Deep SWI involves muscle, bone and/or mediastinum; it must have one of the following conditions: (A) wound opened with the excision of the tissue (I&D), (B) positive culture, and (C) treatment with antibiotics.

The patients were categorized as those without SWI (n = 9157, Group 1) and those with SWI (n = 44, Group 2). The patients with SWI were divided into two groups in accordance with the Centers for Disease Control and Prevention criteria [12]: deep SWI (n = 21) and superficial SWI (n = 23). Immediately after suspicion of SWI, antibiotics were administrated and surgical intervention was made.

The patients' data included the following variables: age, sex, body mass index (BMI), smoking, hypercholesterolemia (whether the patient had a history of hypercholesterolemia diagnosed and/or treated by a physician and/or patient had been assured previously of (a) TG > 200, (b) LDL ≥ 130, (c) HDL < 30, and (d) admission cholesterol > 200 mg/dl), hypertension, peripheral vascular disease (whether the patient had peripheral vascular disease as indicated by claudication either on exertion or at rest, amputation for arterial insufficiency, aorto-iliac occlusive disease reconstruction, peripheral vascular bypass surgery, angioplasty, or stent), diabetes mellitus (defined as a history of diabetes regardless of the duration of disease or need for anti-diabetic agents), length of preoperative hospital stay, functional class according to the Canadian Cardiovascular Society (CCS) [14], and left ventricular ejection fraction (LVEF).

The operation data consisted of the use of cardiopulmonary bypass (CBP), aortic cross-clamp time, number of grafts, and use of double mammary. The postoperative data were comprised of in-hospital mortality (mortality within a 30-day period after operation), utilization of flap or rewiring, interval time between surgery and mediastintis occurrence, re-exploration for bleeding, most common types of microorganism in wound or blood culture, and prolonged intubation time (more than 48 h).

There were three prophylactic antibiotic regimens (only Cefazolin, Cefazolin + Gentamycin, and Cefazolin + Amikacin). All the prophylactic antibiotics were administered as a single dose 30 minutes before surgery and continued up to 48 hours after CABG at 3-hour intervals. Cefazolin was given 1 gr. every 8 hours; those with a weight > 80 received 2 gr. Gentamycin and Amikacin were dispensed 1 mg/kg/dose and 5 mg/kg/dose, respectively. The study protocol was approved by the Ethics Committee of Tehran Heart Center.

### Statistical methods

Numerical variables were presented as mean ± SD, and categorized variables were summarized by percentages. Missing data were present in less than 2.5% of records. Continuous variables were compared using the student's test or nonparametric Mann-Whitney U test whenever the data did not appear to have normal distributions, and categorical variables were compared using the chi-square or Fisher's exact test.

Power analysis showed that was about 69% chance of detecting a significance difference using a two-sided test with significance level = 0.05.

Multivariate forward stepwise logistic regression model for risk factors predicting SWI was constructed. Variables were included into the multivariate model if the p value was found to be less than or equal to 0.15 in the univariate analysis. The associations of independent predictors with SWI in the final model were expressed as odds ratios (OR) with 95% CIs. Model discrimination was measured using the c statistic, which is equal to the area under the ROC curve. Model calibration was estimated using the Hosmer-Lemeshow (HL) goodness-of-fit statistic (higher P values imply that the model fit the observed data better). For the statistical analysis, the statistical software SPSS version 13.0 for windows (SPSS Inc., Chicago, IL) and the statistical package SAS version 9.1 for windows (SAS Institute Inc., Cary, NC, USA) were used. All p values were 2-tailed, with statistical significance defined by p ≤ 0.05.

## Results

Post-CABG SWI occurred in 44 (0.47%) of the 9201 patients. Twenty-three patients (0.24%) had superficial SWI, and 21 patients (0.22%) had deep SWI (bone and mediastinitis). Group 1 (non-SWI) patients were comprised of 74.5% males and 25.5% females at a mean age of 58.5 ± 9.7, while Group 2 (SWI) patients were composed of 47.7% males and 52.3% females at a mean age of 60.1 ± 8.7 (p < 0.001). The mean functional class (Canadian Cardiovascular Society) was 2.1 ± 0.8 in Group 1 (non-SWI) and 2.6 ± 0.7 in Group 2 (SWI) (p < 0.001). Diabetes mellitus (59.1% versus 40.9%) and hypertension (90.9% versus 49.7%) were two significant factors in Groups 2 and 1, respectively (p < 0.001). Also BMI was a significant factor in our study (p = 0.032). On the contrary, some other factors such as cigarette smoking, hypercholesterolemia, preoperative ejection fraction, peripheral vascular disease, and type of antibiotic prophylaxis had no significant impact on the incidence of SWI. These variables are listed in Table 1. Six of the 21 deep SWI patients underwent rotated muscle flap, and the remaining 15 were treated with irrigation and rewiring.

**Table 1:** Pre-intra-and postoperative characteristics among patients with and without SWI

**Variables**	Group 1* **(n = 9157)**	Group 2** **(n = 44)**	**P value**
Age (mean ± SD)	58.5 ± 9.7	60.1 ± 8.7	0.173
BMI (mean ± SD)	27 ± 4	28.3 ± 4	0.032
Female %	25.5	52.3	< 0.001
Smoking %	39.5	34.1	0.467
Diabetes %	33.7	59.1	< 0.001
Hypercholesterolemia %	61	65.9	0.508
Hypertension %	49.7	90.9	< 0.001
PVD %	1.8	2.8	0.666
Length of preoperative hospital stay (days) (mean ± SD)	8.2 ± 4.9	9.4 ± 6.1	0.098
CCS (Functional class) (mean ± SD)	2.1 ± 0.8	2.6 ± 0.7	< 0.001
LVEF (mean ± SD)	49.1 ± 10.2	50.7 ± 11.2	0.260
Graft number (mean ± SD)	3.6 ± 0.9	3.6 ± 1	0.928
Cross clamp time (minute) (mean ± SD)	42.4 ± 41.5	45 ± 16.4	0.686
Perfusion time (minute) (mean ± SD)	70.1 ± 26	75.2 ± 23.5	0.207
Re-exploration for bleeding	1%	13.6%	< 0.001
Intubation time (hours) (mean ± SD)	8.9 ± 13.6	54.1 ± 172.1	< 0.001

Body mass index, BMI; Peripheral vascular disease, PVD; Canadian Cardiovascular Society classification, CCS; left ventricular ejection fraction, LVEF

* Without sternal wound infection

** With sternal wound infection

The in-hospital mortality rates (mortality within a 30-day period after operation) in Group 1(non-SWI) and Group 2 (SWI) were 1.1% and 9.1%, respectively (p < 0.001). The deep SWI patients had a perioperative mortality rate of 14%. There was a significant difference in the rate of re-exploration for bleeding and prolonged intubation time between Group 2 (SWI) and Group 1 (non-SWI) (p < 0.001). In the multivariate analysis, hypertension, re-exploration for bleeding, and the female gender were significant in the development of SWI with a respective odds ratio of 10.7, 13.4, and 2.7 (p ≤ 0.05) (Table 2). The final model had good discrimination (C statistic, 0.827; 95% CI, 0.796–0.859) and calibration (Hosmer-Lemeshow statistic, 17.354; P = 0.015). Positive sternal culture was seen in 72.1% of Group 2 (SWI). In 67.7% of Group 2 (SWI), staphylococcus aureus, 15% of which was Meticilline resistance, was detected as the most common cause. Other microorganisms in order of frequency after S. aureus were staphylococcus epidermidis (6.5%), pseudomonas aeroginosa (6.5%), and streptococcus pneumonia (6.5%). Blood culture was positive with S. aureus as the most common cause in Group 2 (SWI) (total rates of positive blood culture were 14% and 25% in superficial SWI and deep SWI groups, respectively). The distribution of the 21 deep SWI (bone and mediastinitis) cases according to the Oakley classification was as follows: 6 patients in type IIIa, 6 in type IIIb, 8 in IVb, and only one in type V. There were no cases of type I or II; in other words, all the deep SWI cases had risk factors for the development of infection [2]. Tracheostomy was done in 2 patients: one from the superficial SWI group and the other from the deep SWI group. Double internal thoracic arteries were used in none of the SWI cases. The effects of three prophylactic antibiotic regimens (only Cefazolin, Cefazolin + Gentamycin, and Cefazolin + Amikacin) on the development of SWI were also compared. The rates of SWI were relatively similar between these three groups.

**Table 2:** Results of multivariate analysis in development of SWI

**Variables**	**Odds ratio**	95% CI*	**P value**
Re-exploration for bleeding	13.415	4.521–39.802	< 0.0001
Hypertension	10.763	3.297–35.128	< 0.0001
Female	2.707	1.446–5.071	0.0019

* Confidence Interval

## Discussion

Complicating about 3.5% of CABG procedures [4], sternal wound infection is categorized as superficial SWI and deep SWI.

The rate of SWI varies in different reports. Softah et al. reported 2.5% superficial and 0.37% deep sternal wound infection among 3200 patients [5].

In the present study, the respective incidence of superficial and deep SWI was about 0.5% and about 0.22%, which was relatively low in comparison with that in other reports [2-6]

72.1% of our SWI had positive wound culture, with S. aureus being the most common cause in both superficial and deep SWI, which chimes in with the results of similar research in the literature (33–70%) [4,5,7,8]. Positive blood culture was significantly higher in deep SWI than superficial SWI in our study (25% versus 4.3%, respectively).

From a total of 17 evaluated factors in our univariate analysis, 7 factors had a significant positive effect (P < 0.05) in the development of SWI: (a) sex (female gender), (b) preoperative hypertension, (c) high functional class, (d) prolonged intubation time (more than 48 h), (e) diabetes mellitus, (f) re-exploration for bleeding and (g) obesity. The 3 factors of hypertension, female gender, and re-exploration for bleeding were also significant in the multivariate analysis. It is deserving of note that hypertension and the female gender are not viewed as significant risk factors by other related investigations, which necessitates further research for elucidation.

Interestingly, the factors that other studies deem significant, namely cigarette smoking, pump time, and age [5,7,9], had no influence on SWI in our patients.

We compared the effect of three different prophylactic antibiotic regimens which were routinely used in our hospital (only Cefazolin, Cefazolin + Gentamycin, and Cefazolin + Amikacin) on the development of SWI. All these prophylactic antibiotics were given as a single dose preoperatively and continued up to 48 hours postoperatively. The type of antibiotic therapy was in accordance with the preference of the surgeons. The results showed no significant difference between the three groups, and these findings are compatible with other studies [10].

The addition of aminoglycoside to Cefazolin failed to reduce the rate of SWI. We, therefore, employed only Cefazolin as the prophylactic antibiotic (one dose preoperatively, which was continued for 24 hours after operation).

The above-mentioned result holds true for both superficial and deep SWI.

The perioperative mortality rate of our patients was 9.1% for Group 2 (SWI) versus 1.1% for Group 1 (non-SWI), which is relatively similar to that in other reports [7,8]. The perioperative mortality rate in our deep SWI group was 14%; it is an acceptable rate judging by other reports.

In Tehran Heart Center, deep SWI is managed by irrigation and rewiring (if possible) or pectoralis muscle flap in proportion to the condition of the patients and their wounds. There was no one in the Oakley classification of type I or II, for all the causes of deep SWI had one or more risk factors for the development of mediastinitis. Nonetheless, we managed most of our cases (mediastinitis) with irrigation and rewiring [2].

Our use of intracutaneous suture techniques for skin closure (subcuticular suture) in all the cases precluded a comparison between the rates of SWI in the 2 techniques of subcuticular and transcutaneous (mattress suture technique). This comparison was made by Ozalp Karabay, who demonstrated an increased rate of superficial SWI when the intracutaneous suture technique was used relative to the transcutaneous method (16% versus 2%, respectively) [11].

We are inclined to believe that the low rate of SWI in our center is due to a meticulous preoperative, operative, and postoperative care of patients. During the period study (2002–2006), we witnessed no significant fluctuations in the annual rate of SWI. We suggest that a single broad-spectrum antibiotic (Cefazolin) be utilized as a prophylactic antibiotic in CABG patients without the addition of aminoglycoside.

## Conclusion

We studied more than 9000 cases of CABG in order to evaluate multiple risk factors in the development of SWI. Only 3 factors of the female sex, hypertension, and re-exploration for bleeding were significant in our multivariate analysis. The respective rates of superficial SWI and deep SWI were 0.47% and 0.22%, with the latter having a mortality rate of 14%. The addition of aminoglycoside to the prophylactic antibiotic (Cefazolin) had no effect in decreasing the rate of SWI. A host of factors, ranging from lowering the waiting days before surgery, control of the sterility chain, and good hand washing to methodical preoperative, operative, and postoperative care, contributed to our relatively good results.

## List of abbreviations

SWI, Sternal Wound Infection; deep SWI, Deep Sternal Wound Infection; superficial SWI, Superficial Sternal Wound Infection; CABG, Coronary Artery Bypass Grafting; CCS, Canadian Cardiovascular Society classification; CBP, Cardiopulmonary Bypass; CI, Confidence Interval; BMI, Body Mass Index; PVD, Peripheral Vascular Disease; LVEF, Left Ventricular Ejection Fraction

## Competing interests

The author(s) declare that they have no competing interests.

## Authors' contributions

**AS **carried out the surgery and design the study and help to draft manuscript. **AK **participated in the writing the first draft and performed the statistical analysis. **HA **and **SD **and **MM **gave critical comments on the results and participated and design in the revising the manuscript. **NM **and **KA **carried out the surgery. **MB **and **SD **participated in the design of the study and revised the manuscript. **NM **writing the first draft of the manuscript and participated in the performing the statistical analysis. All authors read and approved the final manuscript.

## Pre-publication history

The pre-publication history for this paper can be accessed here:


